# Food Particle Aspiration Associated with Hemorrhagic Shock: A Diagnostic Dilemma

**DOI:** 10.1155/2015/275497

**Published:** 2015-05-19

**Authors:** Basheer Tashtoush, Jonathan Schroeder, Roya Memarpour, Eduardo Oliveira, Michael Medina, Anas Hadeh, Jose Ramirez, Laurence Smolley

**Affiliations:** ^1^Department of Pulmonary and Critical Care Medicine, Cleveland Clinic Florida, Weston, FL, USA; ^2^Department of Otolaryngology, Cleveland Clinic Florida, Weston, FL, USA

## Abstract

The hemodynamic compromise caused by a large aspirated food particle in the airway can become the focus of medical attention and a distraction from rare but fatal Heimlich maneuver related injuries after an incident of food aspiration. We herein present a case of an 84-year-old man who was brought to the emergency department after an episode of choking at a restaurant followed by several failed Heimlich maneuver attempts. Despite relieving the airway obstruction by extracting a large piece of steak from the airway, the patient remained hypotensive and required continued hemodynamic support. Repeated laboratory tests within 24 hrs of aspiration showed a significant decline in the hemoglobin level. A computed tomography (CT) scan of the abdomen and pelvis showed a lacerated liver with a large subcapsular hematoma draining into the pelvis. *Conclusion*. Hepatic rupture is a rare complication of Heimlich maneuver; this paper represents the second case report in the literature. It emphasizes the necessity of early identification and surveillance of fatal Heimlich maneuver complications in a high risk population.

## 1. Case Presentation

An 84-year-old Caucasian man was brought to the emergency department (ED) after an episode of choking at a restaurant. Reports from the emergency medical services (EMS) personnel, who responded to the scene, indicated that the patient had developed signs of choking when a bystander performed several unsuccessful attempts of Heimlich maneuver (HM). Upon EMS arrival, the patient was found to be unresponsive, apneic, and pulseless. Cardiopulmonary resuscitation (CPR) was performed with return of spontaneous circulation after two minutes of CPR. He was intubated in the field by EMS personnel, who reported difficulty during intubation, as an obstructing foreign body prevented proper advancement of the ET tube (size 7) into the trachea.

**Figure 1:**
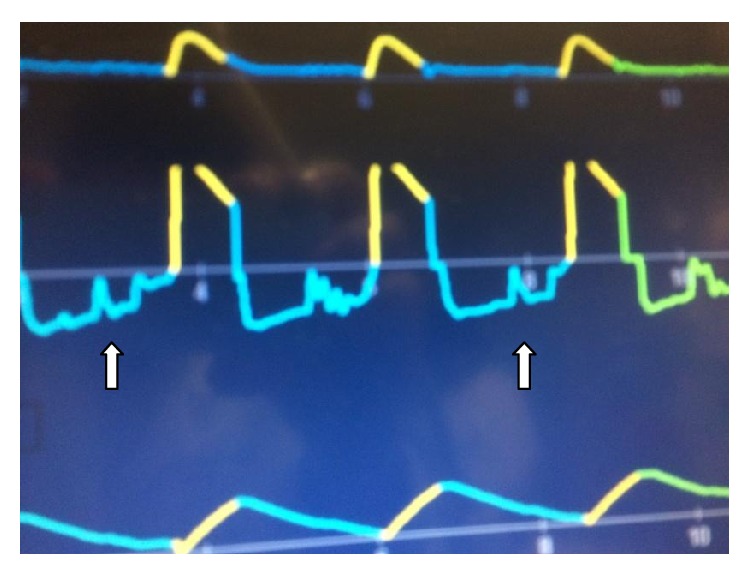
Flow/Time curve on the mechanical ventilator showing auto-PEEP with a notched expiratory flow curve appearing as an “inverted square root sign” (arrows); this represents a complete occlusion of the airway during expiration when the point of equalization of pressures reaches the level of airway obstruction.

Upon arrival to the ED, the patient was found to be hypotensive, requiring immediate fluid resuscitation, vasopressors, and continued mechanical ventilation. He had signs of a persistent large food particle in the main airway as evidenced on the Flow/Time monitor, which showed auto-PEEP with a notched expiratory flow curve, an elevated peak pressure of 53 cm H_2_O, and plateau pressure of 26 cm H_2_O (Figure 1).

**Figure 2:**
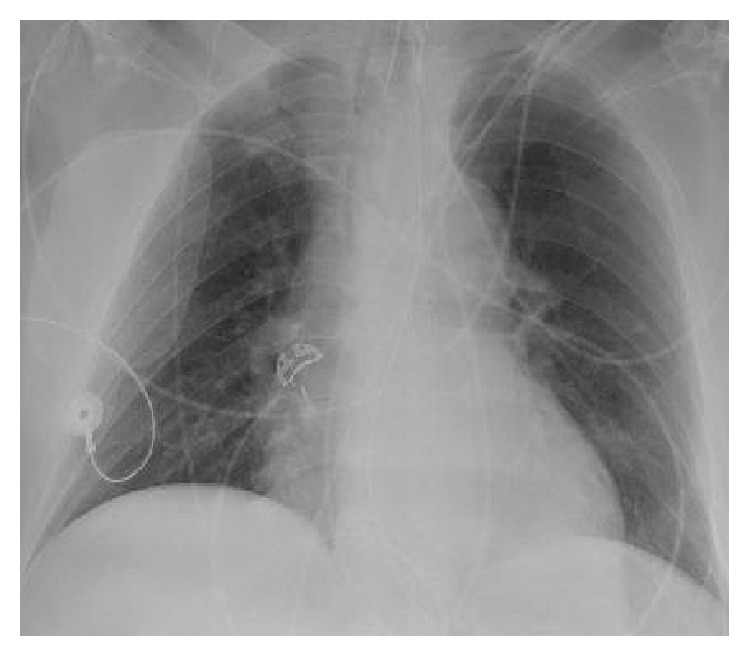
Chest X-ray. Inflated lungs with right upper lobe opacity and a high endotracheal tube position caused by a foreign body in the central airway.

Chest X-ray (Figure 2) showed inflated lungs with a right upper lobe opacity. Empiric antimicrobial treatment for aspiration pneumonia was initiated. A flexible bronchoscopy procedure to extract the food particle was attempted on admission but was unsuccessful, as the forceps could not properly grasp the meat particle without biting through it.

Over the following 12 hours, the patient developed severe refractory shock with multiorgan failure, despite adequate oxygenation. Laboratory test results (Table 1) revealed severe lactic acidosis, acute renal failure, and elevated liver enzymes.

After 24 hours of his initial presentation, the patient was transferred to our facility for rigid bronchoscopy and further management.

Upon arrival, the patient was found to be in severe shock, with a blood pressure of 60/49 mmHg, a heart rate of 150 bpm, and severe lactic acidosis, on maximum dose of IV norepinephrine and vasopressin.

**Figure 3:**
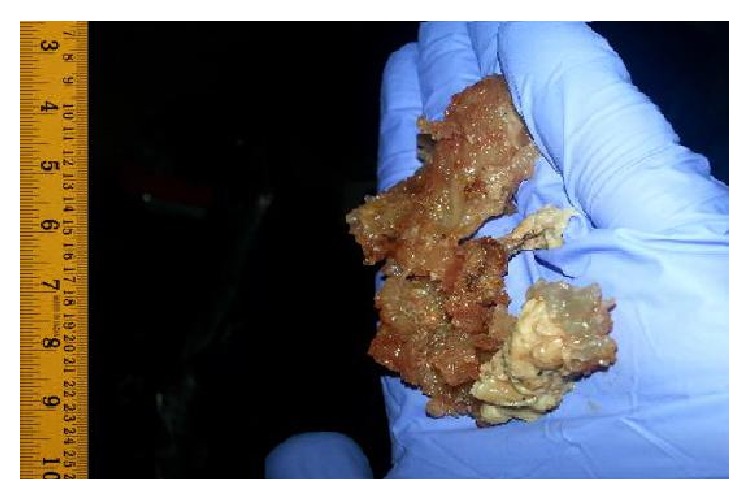
A large piece of steak removed from the trachea with rigid bronchoscopy, approximately 10 cm long.

Rigid bronchoscopy was performed immediately, where the patient was taken to the operating room and placed in neck extended, supine position. A 12 mm external diameter rigid bronchoscope was utilized during the procedure to visualize the trachea and mainstem bronchi while being ventilated via an external side port. A large piece of meat was seen lodged in the distal trachea and extended into the right mainstem bronchus, which was successfully extracted with a grasping crocodile-jaw forceps (Figure 3).

**Figure 4:**
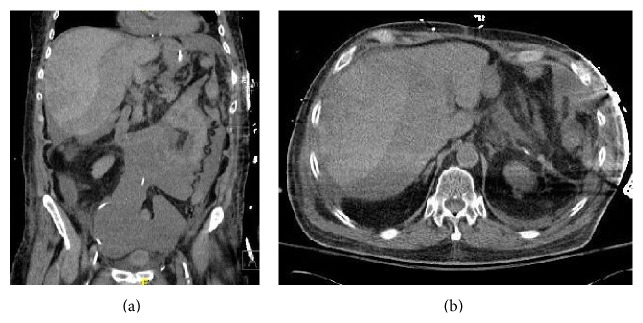
CT abdomen and pelvis. (a) Coronal view and (b) axial view, showing a liver laceration with a large subcapsular hematoma and hemoperitoneum.

Despite relieving the airway obstruction, the patient remained hypotensive and required continued hemodynamic support after the procedure. Repeated laboratory tests drawn upon arrival to our facility showed a significant decline in the hemoglobin level (~3 gm/dL) compared to 24 hours earlier (Table 1). With no obvious external bleed, an intra-abdominal or retroperitoneal hemorrhage was suspected, and immediate transfusion of packed RBCs with fresh frozen plasma was initiated. A CT scan of the abdomen and pelvis (Figure 4) revealed a lacerated liver with a large subcapsular hematoma draining into the pelvis. In the absence of any rib fractures or prolonged CPR, the laceration was attributed to the repeated HM (abdominal thrusts) performed.

Upon further inquiry, family members who witnessed the bystander performing the HM stated that more than ten abdominal thrusts were performed while the patient was in a seated position, as he was unable to stand.

The liver laceration was managed conservatively with transfusion of blood products and close monitoring. Over the following two days, the patient was weaned off vasopressors, and extubated on day three of admission to our facility. Liver enzymes trended down over the course of hospitalization, and the patient was discharged to a rehab facility three weeks later for extended nursing care and physical therapy.

## 2. Discussion

The Heimlich maneuver (HM) was first publicized by Heimlich in 1975 [1]. A year later, the National Research Council reported that 500 lives were saved by the HM [2]. Since that time, vigorous promotion of this technique has saved the lives of many choking victims and became widely accepted as the universal method for relieving foreign body upper airway obstruction [3]. However, several rare but life threatening complications have been reported from properly and improperly performed HM.

Complications such as rib and vertebral fractures, retinal detachment [4], diaphragmatic rupture [5], ruptured aortic valve [6], acute thrombosis of abdominal aortic aneurysm [7–10], aortic stent-graft displacement [11], mesenteric laceration [12], and many other fatal traumatic injuries of the gastrointestinal tract have also been reported [13–18].

Hepatic rupture is an extremely rare complication of HM with only one previously reported case in literature in 2007 by Palleiro et al. [19].

Our patient's family members stated that a Good Samaritan performed the maneuver more than ten times while the patient was in a seated position. This may have contributed to the hepatic injury, as the correct application of HM in a seated victim has not been well described, and abdominal thrusts may have been directly applied to the liver.

Common features shared with the case reported by Palleiro et al. include patients age, both in their 80s, and meat aspiration occurred in both cases. However, there was no precise description of how the HM was performed in the previously reported case.

Even when performed correctly, the maneuver can be associated with rare complications. Rib fractures and gastric and esophageal perforations are among the most frequently reported [20, 21].

In this case, the large piece of meat in the airway performed as a ball valve, demonstrating auto-PEEP on the Flow/Time curve of the mechanical ventilator monitor (Figure 1), with a notched expiratory flow curve giving the “inverted square root sign,” where the particle completely but transiently occludes the expiratory airflow towards the end of expiration when the point of equalization of pressures reaches the level of obstruction. After this transient flow cessation, expiratory airflow resumes as the point of equalization of pressures passes the level of obstruction.

Auto-PEEP can cause significant hemodynamic compromise and circulatory collapse, as the increased intrathoracic pressure associated with auto-PEEP reduces venous return (preload) and increases pulmonary vascular resistance, precipitating right ventricular strain and failure. In addition, auto-PEEP can augment left ventricular afterload due to increased negative intrapleural pressure generated by the patient when he/she triggers the ventilator. All these phenomena can severely reduce the cardiac output and precipitate shock [22, 23].

Urgent rigid bronchoscopy in a hemodynamically unstable patient with central airway obstruction is critical. In this patient the delay in performing rigid bronchoscopy occurred due to the flexible bronchoscopy attempt and the lack of resources for rigid bronchoscopy at the referring facility, in addition to the patient's hemodynamic instability, which delayed the decision to transfer the patient.

Because of the major airway obstruction in our patient, the auto-PEEP was thought to be the primary cause of the persistent hypotension and circulatory failure. However, when the obstruction was removed, the persistent shock and the decline in the hemoglobin level indicated that a concealed source of hemorrhage was overlooked.

Given the potential for critical abdominal organ injuries in association with the HM, there appears to be an emerging necessity to establish surveillance guidelines for internal organ injuries when evaluating high risk patients who undergo HM, for example, children, the elderly, and patients with impaired consciousness.

## 3. Conclusion

Hepatic rupture is a rare complication of Heimlich maneuver; this paper represents the second case report in the literature. It emphasizes the necessity of early identification and surveillance of fatal Heimlich maneuver complications in a high risk population.

## Figures and Tables

**Table 1:** Laboratory test results on admission and repeated tests 24 hours later.

Laboratory tests on arrival to ED	Laboratory tests 24 hrs after aspiration
Arterial blood gas analysis (ABG)	
PH: 7.21	PH: 7.38
PCO_2_: 28 mmHg	PCO_2_: 32 mmHg
PO_2_: 423 mmHg	PO_2_: 134 mmHg
HCO_3_: 11.2 mmol/L	HCO_3_: 19.3 mmol/L
Lactate: 12.9 mmol/L	Lactate: 14.34
FiO_2_: 100%	FiO_2_: 60%

Serum chemistry tests	
Glucose: 120 mg/dL	Glucose: 174 mg/dL
K: 3.6 mmol/L	K: 4.9 mmol/L
Na: 143 mmol/L	Na: 148 mmol/L
Cl: 117 mmol/L	Cl: 105 mmol/L
HCO_3_: 12 mmol/L	HCO_3_: 22 mmol/L
Ca: 7.7 mg/dL	Ca: 8.2 mg/dL
Mg: 2.0 mg/dL	Mg: 2.2 mg/dL
BUN: 31 mg/dL	BUN: 36 mmol/L
Creatinine: 2.0 mg/dL	Creatinine: 2.1 mg/dL
ALB: 2.5 gm/dL	ALB: 2.5 gm/dL
TPROT: 4 gm/dL	TPROT: 3.8 gm/dL
ALKPHOS: 103 U/L	ALKPHOS: 156 U/L
ALT: 426 U/L	ALT: 3947 U/L
AST: 2307 U/L	AST: >7000 U/L
TBILI: 0.9 mg/dL	TBILI: 1.7 mg/dL

Complete blood count	
WBC: 12.56 k/*μ*L	WBC: 13.09 k/*μ*L
HB: 8.1 g/dL	HB: 5.3 g/L
HCT: 25%	HCT: 16.8%
PLT: 133 k/*μ*L	PLT: 155 k/*μ*L

Coagulation profile	
APTT: 31.0 sec	APTT: 31.8 sec
INR: 1.5	INR: 1.6
